# Identification of a glioma functional network from gene fitness data using machine learning

**DOI:** 10.1111/jcmm.17182

**Published:** 2022-01-19

**Authors:** Chun‐xiang Xiang, Xi‐guo Liu, Da‐quan Zhou, Yi Zhou, Xu Wang, Feng Chen

**Affiliations:** ^1^ Department of Pathology Xiangyang Central Hospital Affiliated Hospital of Hubei University of Arts and Science Xiangyang, Hubei China; ^2^ Department of Head and Neck Surgery Hubei Cancer Hospital Wuhan, Hubei China; ^3^ Department of Neurosurgery Xiangyang Central Hospital Affiliated Hospital of Hubei University of Arts and Science Xiangyang, Hubei China

**Keywords:** co‐functional network, CRISPR‐Cas9, glioma, prognostic biomarkers, scRNA‐seq

## Abstract

Glioblastoma multiforme (GBM) is an aggressive form of brain tumours that remains incurable despite recent advances in clinical treatments. Previous studies have focused on sub‐categorizing patient samples based on clustering various transcriptomic data. While functional genomics data are rapidly accumulating, there exist opportunities to leverage these data to decipher glioma‐associated biomarkers. We sought to implement a systematic approach to integrating data from high throughput CRISPR‐Cas9 screening studies with machine learning algorithms to infer a glioma functional network. We demonstrated the network significantly enriched various biological pathways and may play roles in glioma tumorigenesis. From densely connected glioma functional modules, we further predicted 12 potential Wnt/*β*‐catenin signalling pathway targeted genes, including AARSD1, HOXB5, ITGA6, LRRC71, MED19, MED24, METTL11B, SMARCB1, SMARCE1, TAF6L, TENT5A and ZNF281. Cox regression modelling with these targets was significantly associated with glioma overall survival prognosis. Additionally, TRIB2 was identified as a glioma neoplastic cell marker in single‐cell RNA‐seq of GBM samples. This work establishes novel strategies for constructing functional networks to identify glioma biomarkers for the development of diagnosis and treatment in clinical practice.

## 1 INTRODUCTION

Glioblastoma (GBM) remains the most common and aggressive (Grade IV) central nervous system (CNS) tumour^1^, ^2^ with median overall survival of up to 14–16 months.^3^, ^4^, ^5^ Current GBM treatment regimens constitute a combination of radiotherapy with adjuvant Temozolomide (TMZ) chemotherapy, which could expand the life expectancy by 1.8 years on average.^6^, ^7^ Since prognoses and therapy responses vary dramatically among GBM patients, there remains the need to identify early diagnostic GBM biomarkers. One consensus was recently reached that IDH (Isocitrate Dehydrogenase 1) could be one biomarker based on which GBM can be divided as IDH‐wild type and IDH‐mutant.^2^ The IDH‐wild type tends to affect older people (mean age of 62) as the primary tumour and accounts for most of GBMs (~90%), while the IDH‐mutant presents in the secondary GBM, which progresses from lower‐grade glioma. However, the association between IDH status and GBM prognosis remains poorly understood.

In this study, we proposed a novel strategy to identify biomarkers by constructing a landscape of co‐functional associations in the context of glioma, termed as Glioma Functional Network (GFN). Unlike previously published biological networks, such as protein‐protein interaction networks,^8^ gene co‐expression networks,^9^ the functional networks revealed gene‐gene associations that do not necessarily physically interact or share similar expression patterns.

Initially, functional networks were constructed using double gene knockouts on a genome‐wide scale.^10^ However, this strategy is not feasible in the human genome given that the combination space would increase tremendously, making experimental and computational approaches rather challenging. With recent advances in genome‐wide CRISPR‐Cas9 functional screening, there are accumulating studies^11^, ^12^, ^13^, ^14^ focusing on genome‐wide single‐gene knockouts via CRISPR based techniques, and generating gene fitness data correlating the extent of cell proliferation to gene perturbations. While such data were generated across large pools of cell lines, computing pairwise functional similarities and inference genetic interactions can be implemented. Several studies have demonstrated the functional networks inferred from gene fitness screen data could recapitulate protein complexes^15^ and functional modules.^16^ Given that, there still lacked functional networks focusing on gliomas, we sought to fill the gap by implementing a novel systematic strategy to identify gene co‐functional networks using machine learning algorithms.

## 2 MATERIALS AND METHODS

### 2.1 Predicting glioma functional network from CRISPR screening data using machine learning

**FIGURE 1:**
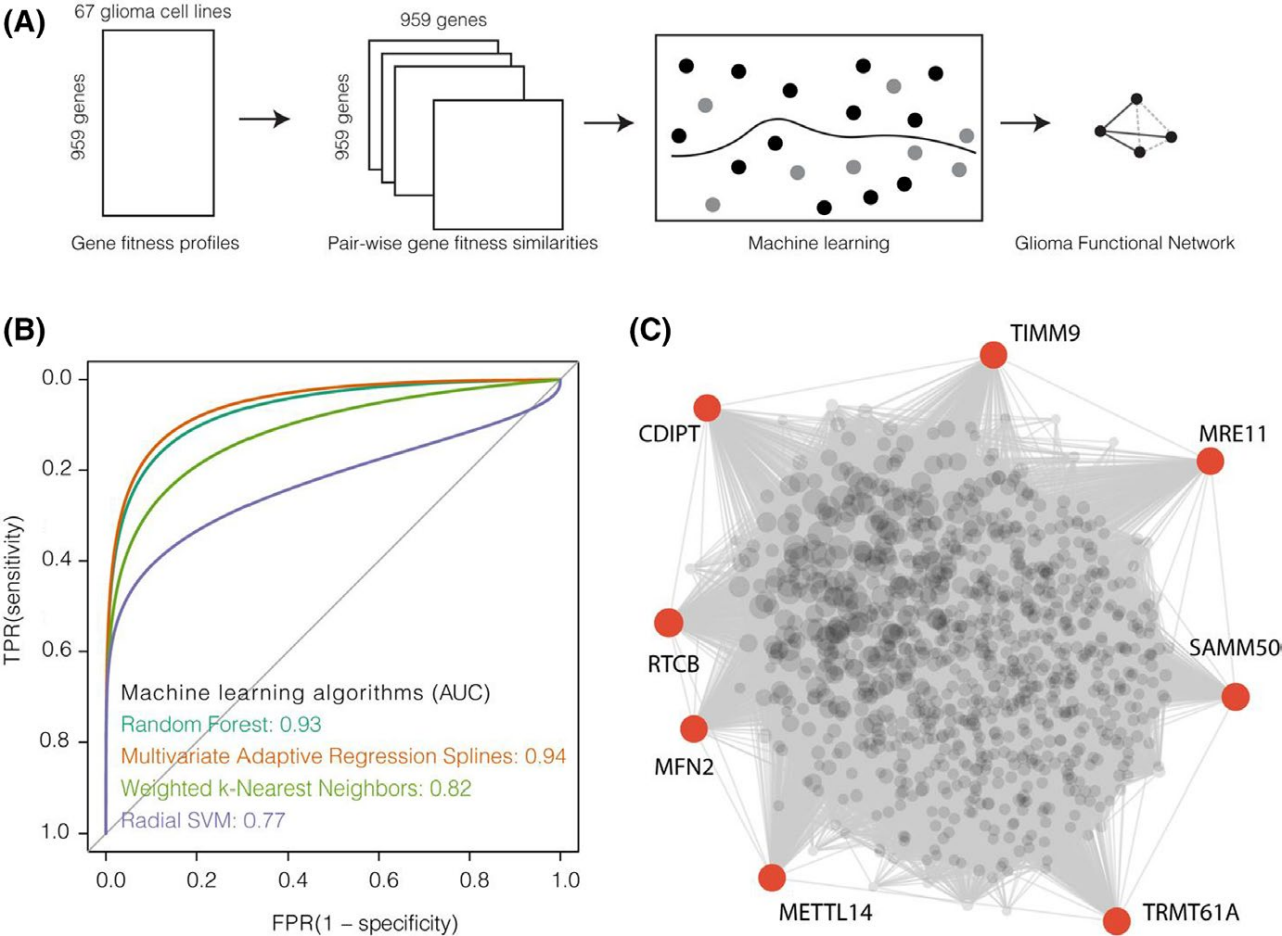
Generation of the glioma functional network. (A) Diagram of the computational framework for generating glioma functional networks. (B) ROC analysis to benchmarking machine learning algorithms for predicting co‐functional gene associations. The AUC (area under curve) values were each algorithm was computed as shown in the brackets. (C) Landscape of the glioma functional network. The size of the node reflects the degree of each node. The grey lines denote predicted functional associations. The identified hub genes were highlighted in red

Gene fitness data (version 21Q3) from CRISPR screening and RNA‐seq expression data were downloaded from the Depmap portal (https://depmap.org/portal/) and CCLE (Cancer Cell Line Encyclopedia, https://portals.broadinstitute.org/ccle) project respectively. To construct glioma specific networks, we retrained data from a total of 67 glioma cell lines (Figure 1A). Several steps were applied to pre‐process the data to select informative genes for predicting the network. Firstly, it was suggested that not all genes are expressed in cell lines, due to the inherent nature of genomic alterations in cancer cells.^15^ Therefore, in each cell line, genes with less than 0 TPM (Transcript Per Million) were eliminated. Then, genes with fitness scores less than −0.5 in the most dependent cell lines were retained, as drastic fitness effects upon genetic depletion would facilitate functional relationships in the network construction. Lastly, genes with high variations in fitness data were retained. The filtration criterion is 1 MAD (median absolute deviation) greater than the population MAD. Finally, a total of 959 genes were selected to prepare the training data (Figure 1A).

To generate feature data as input for the machine learning pipeline, a series of similarity metrics, including Pearson correlation coefficient, Spearman's rank correlation coefficient, Euclidean distances, Dice's coefficient, Manhattan distance, Minkowski distance, Chebyshev distance, Harmonic mean, Jaccard index and mutual information, were computed among 959 genes in pairwise combinations. The R package, philentropy (version 0.5.0)^17^ was used to compute these similarities, and 10 sets of feature data for a total of 459,361 gene pairs were generated (Table S1).

To generate reference data for machine learning, co‐functional gene pairs reported from at least two out of three previous studies^15^ were used as positives. Then, pairwise gene combinations excluding the aforementioned positives and sharing no common Gene Ontology (GO) annotations were considered as negatives. The Bioconductor package, org. Hs.eg.db (version 3.14.0), was used for mapping GO annotations. As a result, a total of 50,481 positives and 3,055,099 negatives were generated as reference data for machine learning.

For the machine learning pipeline, the fivefold cross validation was implemented and repeated 10 times for parameter tuning. Four machine learning algorithms: random forest (RF),^18^ Multivariate Adaptive Regression Splines (MARS),^19^ Support Vector Machines^20^ with Radial Basis Function Kernel (svmRadial) and Weighted *k*‐Nearest Neighbor Classifier (kknn).^21^ The performances of the algorithms were benchmarked using receiver operating characteristic (ROC) analysis. The area under the ROC curves (AUROC) were computed for each algorithm, and the best performances were achieved by the MARS algorithm with AUROC of 0.94 (Figure 1B). The optimal threshold was determined using the coords function from the R package, pROC (version 1.18.0).^22^ A total of 47,475 gene pairs with MARS scores greater than 0.02 were selected as associated interactions for the glioma functional network (Figure 1C).

### 2.2 Detecting glioma functional modules

To detect modules from the glioma functional network, the ClusterONE^23^ algorithm was used to predict modules from the functional network. Briefly, the ClusterONE algorithm aims to increase dense regions from randomly selected genes from the network and then identify groups of high cohesiveness as modules.^23^ For this study, the program was downloaded from the CluterONE website (https://paccanarolab.org/static_content/clusterone/cluster_one‐1.0.jar), and a total of 88 modules were detected from the glioma functional network.

### 2.3 Differential gene expression analysis in gliomas

To identify differentially expressed genes in glioma samples, the microarray intensity files (*.CEL) of three brain tumour studies, including the Repository for Molecular Brain Neoplasia Data (Rembrandt),^24^ were downloaded from the NCBI GEO database with accession numbers: GSE68848 (Rembrandt), GSE16011 ^25^ and GSE50161.^26^ The raw data were normalized using the Bioconductor package, *affy* ^27^ and annotated using the custom CDF files (version: 25).^28^ The Bioconductor package, *limma*,^29^ was used to fit the linear models for differential gene expression analysis by comparing brain tumour and normal healthy samples.

### 2.4 Survival analysis of β‐catenin target genes

For the GBM survival analysis, gene expression and survival data were retrieved from The Cancer Genome Atlas (TCGA) portal (https://portal.gdc.cancer.gov/), Rembrandt and other large cohort studies^30^ from NCBI GEO database with accession numbers: GSE13041,^31^ GSE83294,^30^ GSE16011,^25^ GSE7696 ^32^ and GSE83130.^33^ Expression profiles of *β*‐catenin target genes were extracted and fitted into the Cox proportional hazards regression model^34^ to summarize the prognostic score for each sample (Figure 6) using the following formula:

$$h\left(t\right)=h_{0}\left(t\right)\times exp\left(\sum_{i=1}^{p}b_{i}\times X_{i}\right)$$

where *h*(*t*) is the expected hazard at time *t*, *h*_0_(*t*) is the baseline hazard, *X_i_* represents the expression levels of *β*‐catenin target genes predicted in this study, and *b_i_* is the regression coefficient coefficient for gene *i*. For each cohort (Figure 6), the Cox proportional hazards regression modelling was implemented using the *coxph* function from the R package, *survival*. The *predict*.*coxph* function was used to compute the risk scores. Then, samples were ranked based on the score and divided into two groups with high and low risks with a cut‐off at the median value of the population scores. The significance of the difference of overall survival outcomes was evaluated using log‐rank tests.

### 2.5 GBM single‐cell RNA‐seq data analysis

Single‐cell RNA‐seq of GBM data were retrieved from three independent cohorts including single‐cell suspensions from untreated IDH‐wild type glioblastomas,^35^ IDH‐mutant astrocytomas and oligodendrogliomas.^36^ Fresh tissues were subjected to droplet‐based single‐cell RNA‐seq pipeline. The raw gene counts data were retrieved from NCBI GEO database with accession numbers: GSE89567 and GSE138794, the deposited website (https://github.com/mbourgey/scRNA_GBM). The Bioconductor packages, *scater* and *scran*, were used for data normalization, dimension reduction and clustering. To identify glioma neoplastic cells, the *SingleR* package was used to annotate the cells by correlating gene expression profiles with a previous published study.^37^ Briefly, a total of 3589 cells were sorted from four GBM patients and subjected to RNA‐seq. Using differential gene expression analysis, seven types of cells were identified including astrocytes, immune cells, neoplastic cells, neurons, oligodendrocytes, OPC (oligodendrocyte precursor cells) and vascular cells. The normalized gene counts, cell type assignments and reduced dimension data were downloaded from the website (http://www.gbmseq.org/).

### 2.6 Validation of GFN using published protein‐protein interaction networks and protein complexes

To validate GFN, protein‐protein interaction (PPI) networks were retrieved from previous studies, including InBio_Map,^38^ STRING (version: 11.5)^39^ and BioGRID (version: 4.4.202).^40^ For the STRING data, PPIs were filtered with confidence scores greater than 500. The GO semantic similarities among interacting protein pairs were computed using the Bioconductor, *GOSemSim*, package.^41^ The manually curated complexes were retrieved from the comprehensive resource of mammalian protein complexes (CORUM, version 3.0).^42^

## 3 RESULTS

### 3.1 A glioma functional network (GFN) generated from gene fitness screening data

**FIGURE 2:**
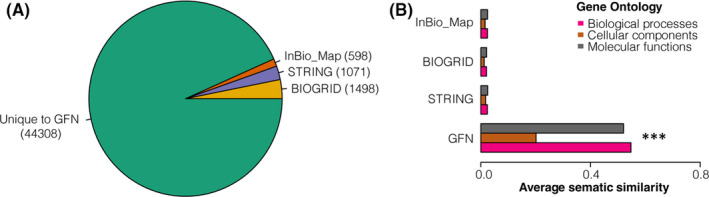
Benchmarking of GFN with published databases, including InBio_Map,^38^ STRING (version: 11.5)^39^ and BioGRID (version: 4.4.202).^40^ (A) Pie chart showing numbers of co‐functional gene pairs published in public databases. (B) Bar graphs showing comparisons of average GO semantic similarities among gene pairs from GFN and other published databases. (***p < 0.001 by Wilcoxon rank sum and signed rank tests)

Genome‐wide CRISPR screening of gene fitness in cancer cell lines has provided abundant data to generate functional networks^43^ and elucidated the landscape of gene regulations in an unprecedented systematic manner. However, methodologies involved in these studies were limited to Pearson's correlation coefficient^43^ or linear modelling.^16^ We sought to implement a novel systematic strategy by incorporating similarity metrics with machine learning approaches to generate functional scores (Figure 1A). After data preprocessing, we first applied ten similarity metrics (see Methods) to pairwise combinations of 959 candidate genes. Then, the resulting feature data were fitted with four machine learning models, including random forest (RF),^18^ Multivariate Adaptive Regression Splines (MARS),^19^ Support Vector Machines^20^ with Radial Basis Function Kernel (svmRadial) and Weighted k‐Nearest Neighbor Classifier (kknn)^21^ for training. We evaluated performances of these algorithms by receiver operating characteristic (ROC) analysis. As shown in Figure 1B, MARS performed better than other algorithms with the largest area under ROC (AUROC) of 0.94. The algorithm aims to ensemble a series of linear models and non‐linear models. Thus, it achieved the best prediction performance. From the ROC analysis, the cut‐off of a score of 0.02 was chosen to identify a total of 47,475 high confident co‐functional associations (Figure 1C, Table S1) as glioma functional networks (GFN) from 459,361 scored gene pairs. At this cut‐off, the machine learning strategy yielded a sensitivity of 0.86 and a specificity of 0.90. As shown in Figure 2A, the majority (93.3%) of the co‐functional associations were not published, while the remaining overlapped with recently published databases, including InBio_Map (598), STRING (1071) and BIOGRID (1498). Although poorly overlapping with existing databases, GFN yielded significantly higher GO semantic similarities of 0.20 in biological processes, 0.52 in cellular components and 0.55 in molecular functions (Figure 2B), which suggested as a novel resource with high biological relevances.

**FIGURE 3:**
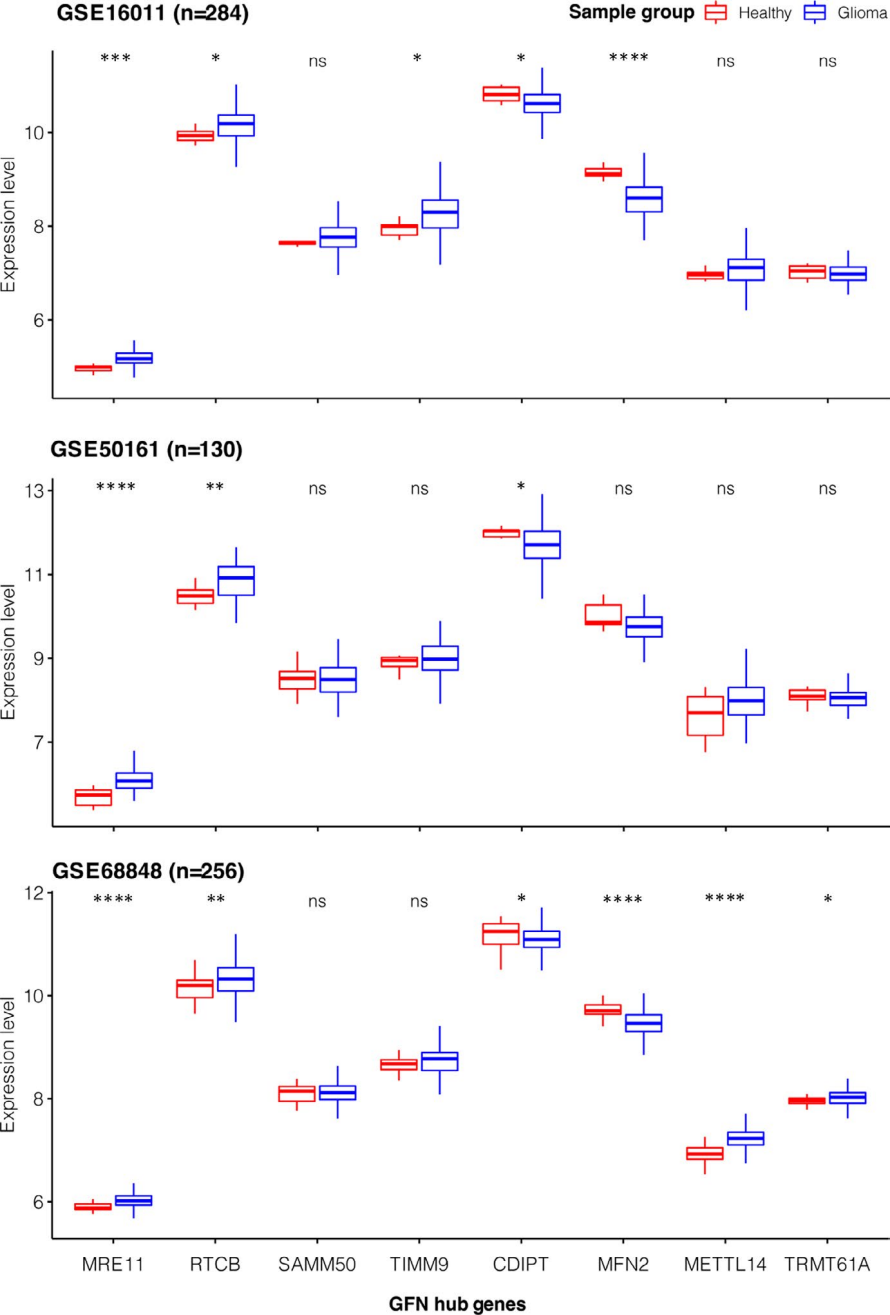
Box plots showing comparison of expression levels of glioma functional network hub genes in normal healthy and glioma samples. Genome‐wide expression profiles were retrieved from three independent studies: GSE68848 (Rembrandt), GSE16011 ^25^ and GSE50161.^26^ (ns, not significant, *p < 0.05; ** *p* < 0.01; ***p < 0.0001, ****p < 0.00001,by Wilcoxon rank sum and signed rank tests)

We then hypothesised that GFN may involve the pathogenesis of gliomas. Test this, each gene in the GFN was ranked by the Kleinberg's hub centrality scores,^44^ and top 8 genes, which included RTCB (RNA 2’,3’‐Cyclic Phosphate And 5’‐OH Ligase), SAMM50 (sorting and assembly machinery component), TRMT61A (tRNA methyltransferase 61A), MRE11 (double‐strand break repair nuclease), METTL14 (Methyltransferase 14, N6‐adenosine‐methyltransferase subunit), MFN2 (Mitofusin 2), CDIPT (CDP‐diacylglycerol‐inositol 3‐phosphatidyltransferase) and TIMM9 (Translocase of inner mitochondrial membrane 9), were identified as GFN hub genes (Figure 1C). Six of the eight hub genes exhibited consistent patterns in the changes of expression levels across three independent cohorts of glioma samples (Figure 3). MRE11, RTCB, TIMM9 and METTL14 were up‐regulated in gliomas, while CDPIT and MFN2 were down‐regulated. MRE11 is engaged in DNA damage repair pathways, and it was previously reported to be involved in the breast cancer progression,^45^ and played a role in the response of drug treatment in glioma.^46^ METTL14 promoted differentiation of embryonic stem cells^47^ and may regulate genes involved in cell proliferation, differentiation and DNA damage.^48^ On the contrary, MFN2 was known as a tumour suppressor and exhibited lower expression in cancers.^49^ Taken together, as central players in the network, dysfunctions of these genes would suggest GFN identified from this study played roles in glioma tumorigenesis.

### 3.2 GFN modules significantly enriched in biological pathways

**FIGURE 4:**
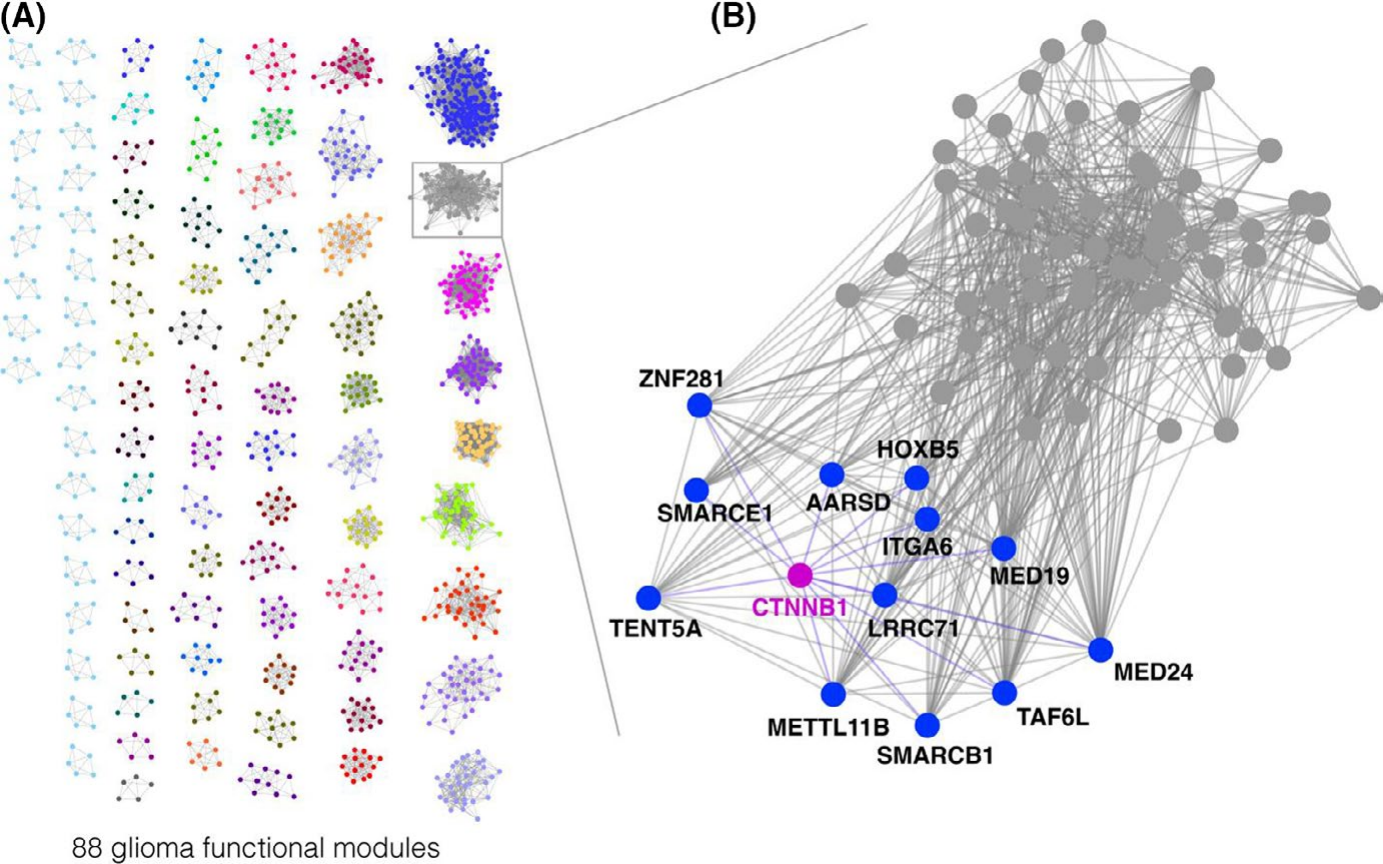
Generation of glioma functional communities. (A) Overview of 88 identified glioma functional communities. (B) Zoomed in view of the functional community consisting of *β*‐catenin (magenta) and its predicted targets (blue) based on co‐functional associations

We next implemented a clustering algorithm, ClusterOne, to identify a total of 88 functional modules from GFN (Figure 4A, Table S2), which consisted of a range of 5 to 212 members. Gene set enrichment analysis revealed that the glioma functional modules significantly enriched in biological pathways, including aminoacyl tRNA biosynthesis (CID‐02, *p* = 1.83 × 10^−11^), Terpenoid backbone biosynthesis (CID‐06, *p* = 5.59 × 10^−3^), vibrio cholerae infection (CID‐16, *p* = 7.06 × 10^−10^) and soluble *N*‐ethylmaleimide‐sensitive factor attachment protein receptor (SNARE) interactions in vesicular transport (CID‐32, *p* = 4.40 × 10^−3^). Aminoacyl‐tRNA biosynthesis involved in the various biological functions, including immune regulation.^50^ It also played roles in neurodegenerative disease,^51^ and pontocerebellar hypoplasia.^52^ Deregulations of pathway members including AIMP1, AIMP2 and AIMP3 were observed in gastric and colorectal cancer.^53^ One previous study showed that the Terpenoid backbone biosynthesis pathway was down‐regulated in glioblastoma cells due to the knock‐down of lncRNA HULC, which was involved in cell proliferation.^54^ Glioma progression associated genes identified from clustering and differential expression analysis were significantly enriched in the vibrio cholerae infection pathway.^55^ The SNARE interactions in vesicular transport involved in the fusion of multivesicular body and cell membranes.^56^ Knockdown of one of SNARE proteins, Stx1, could inhibit cell growth and invasion in glioblastoma.^57^ In summary, pathway analysis revealed the GFN modules significantly enriched in glioma tumorigenesis, which could assist investigating glioblastoma biology.

Previous studies demonstrated that co‐functional networks could recapulated protein complexes.^15^ Consistent with these findings, GFN modules were also significantly overlapped with protein complexes, including 55S mitochondrial ribosome (CID‐02, *p* = 1.83 × 10^−11^), origin recognition complex (CID‐05, *p* = 7.44 × 10^−4^), PPP2CA‐PPP2R1A complex (CID‐06, *p* = 1.61 × 10^−3^), Condensin II (CID‐08, *p* = 1.05 × 10^−3^), ITGA3‐ITGB1‐BSG complex (CID‐11, *p* = 6.43 × 10^−4^), Rnase/Mrp complex (CID‐12, *p* = 4.84 × 10^−5^), Spliceosome (CID‐13, *p* = 7.30 × 10^−4^; CID‐27, *p *= 7.67 × 10^−4^), Mediator complex (CID‐14, *p* = 6.65 × 10^−4^), v‐ATPase‐Ragulator‐AXIN/LKB1‐AMPK complex (CID‐16, *p* = 8.35 × 10^−3^), CENP‐A‐histone H4 heterodimer‐HJURP complex (CID‐23, *p* = 4.45 × 10^−5^), eIF3 complex (CID‐26, *p* = 1.94 × 10^−4^), MYC‐MAX complex (CID‐39, *p* = 1.21 × 10^−5^), Prefoldin complex (CID‐49, *p* = 4.57 × 10^−4^), RAD6A‐KCMF1‐UBR4 complex (CID‐50, *p* = 3.96 × 10^−03^), RAD51B‐RAD51C‐RAD51D‐XRCC2‐XRCC3 complex (CID‐62, *p* = 7.67 × 10^−6^), RAD51C‐XRCC3 complex (CID‐73, *p* = 9.08 × 10^−6^) and 20S proteasome (CID‐83, *p* = 3.61 × 10^−4^). As proteins tend to interact as complexes to carry out functions, GFN modules provide an extra layer of information to better understand various roles in the underlying biology of glioma pathogenesis.

### 3.3 Prediction of β‐catenin targets from glioma functional modules

Accumulating evidence suggests that one of the embryonic stem cell signalling pathways, Wnt/*β*‐catenin pathway, is involved in the proliferation^58^, ^59^ and prognosis^60^ of gliomas, which prompts this pathway as potential therapeutic target.^61^ Therefore, *β*‐catenin dysregulations served as a hallmark in cancer progression.^62^ Alongside with the concept of glioma stem cells (GSCs), the Wnt/*β*‐catenin pathway has gained interest from the research community in recent decades.^63^ Thus, we sought to further predict *β*‐catenin potential targets inferred by the glioma functional modules, since the regulator and its targets may be functionally associated. We identified that *β*‐catenin is among the members of module CID‐02 (Figure 4B) and was functionally associated with 12 genes including AARSD1 (Alanyl‐tRNA synthetase domain containing 1), HOXB5 (Homeobox B5), ITGA6 (Integrin subunit Alpha 6), LRRC71 (Leucine‐rich repeat containing 71), MED19 (Mediator complex subunit 19), MED24 (Mediator complex subunit 24), METTL11B (N‐Terminal Xaa‐Pro‐Lys *N*‐methyltransferase 2), SMARCB1 (SWI/SNF‐related matrix‐associated actin‐dependent regulator of chromatin subfamily B member 1), SMARCE1 (SWI/SNF‐related, matrix‐associated, actin‐dependent regulator of chromatin, subfamily E, member 1), TAF6L (TATA‐box binding protein associated factor 6 like), TENT5A (Terminal nucleotidyltransferase 5A) and ZNF281 (Zinc finger protein 281) (Table 1). Some of predicted targets are in line with previous studies, as Wnt/*β*‐catenin pathway has potential affect in mediator complexes and SWI/SNF complexes.^64^ Additionally, HOXB5 was one of homeobox genes and interacted conservely with Wnt/*β*‐catenin pathway.^65^ Experimental data suggested that HOXB5 involved in the progression of breast cancer through Wnt/*β*‐catenin pathway.^66^ In summary, various pieces of evidence suggested biological functions of *β*‐catenin targets, which could play roles in glioma pathogenesis.

**TABLE 1:** List of 12 predicted *β*‐catenin targets

Gene Symbol	GeneID	Chromosome location	Description
AARSD1	80755	17q21.31	Alanyl‐tRNA synthetase domain containing 1
HOXB5	3215	17q21.32	Homeobox B5
ITGA6	3655	2q31.1	Integrin subunit alpha 6
LRRC71	149499	1q23.1	Leucine rich repeat containing 71
MED19	219541	11q12.1	Mediator complex subunit 19
MED24	9862	17q21.1	Mediator complex subunit 24
METTL11B	149281	1q24.2	*N*‐terminal Xaa‐Pro‐Lys *N*‐methyltransferase 2
SMARCB1	6598	22q11.23|22q11	SWI/SNF related, matrix associated, actin dependent regulator of chromatin, subfamily b, member 1
SMARCE1	6605	17q21.2	SWI/SNF related, matrix associated, actin dependent regulator of chromatin, subfamily e, member 1
TAF6L	10629	11q12.3	TATA‐box binding protein associated factor 6 like
TENT5A	55603	6q14.1	Terminal nucleotidyltransferase 5A
ZNF281	23528	1q32.1	Zinc finger protein 281

**FIGURE 5:**
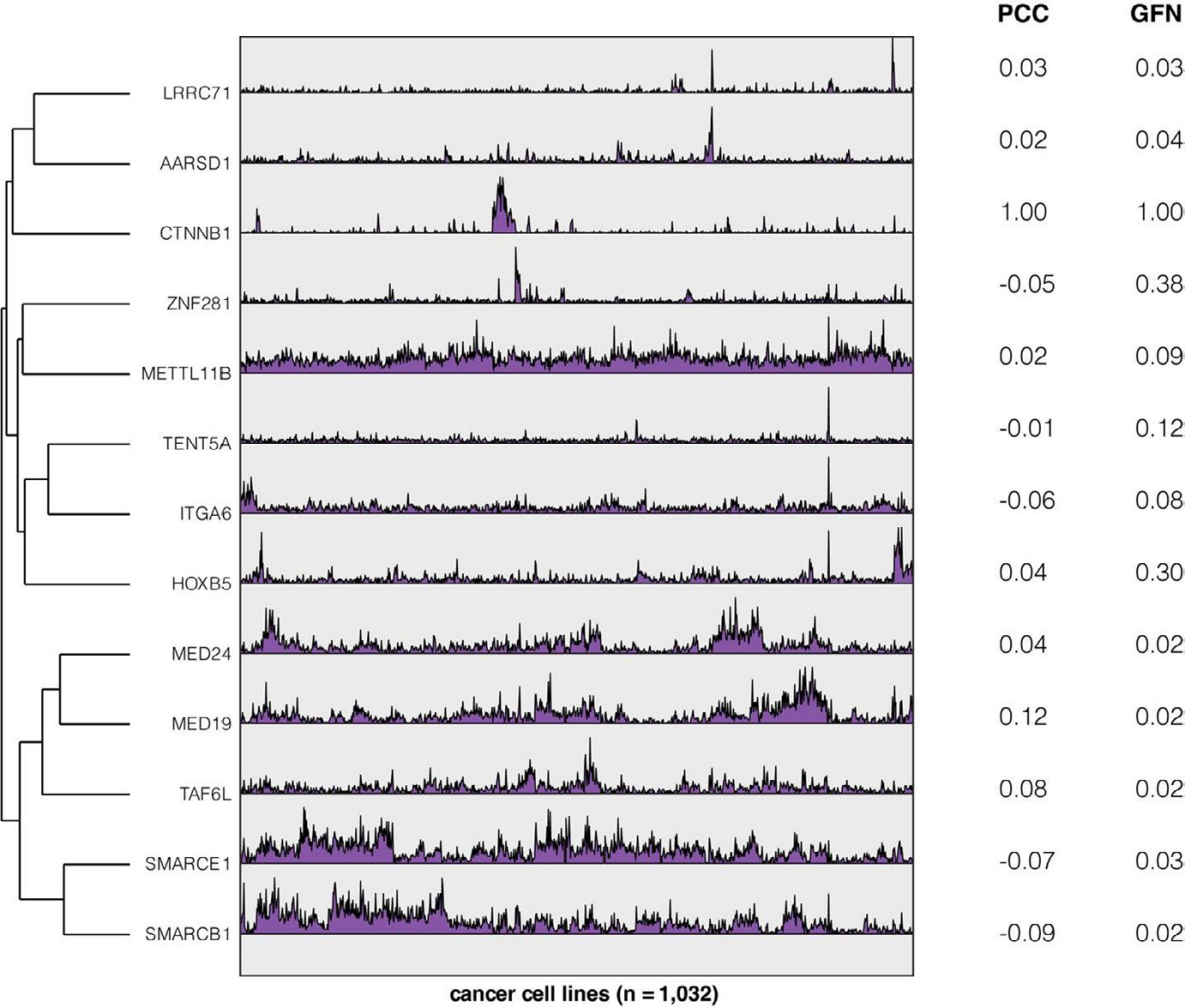
Fitness profiles in cancer cell lines (*n* = 1032) for *β*‐catenin and its predicted targets from the glioma functional modules. Functional associated scores between *β*‐catenin and its predicted targets were computed using PCC and retrieved from the GFN. Rows (genes) are hierarchically clustered based on PCC scores

Notably, we compared predicted scores of *β*‐catenin targets from our study with previously established methods based on Pearson correlation coefficient (PCC). The PCC scores of the predicted targets ranged from −0.06 to 0.12 (Figure 5), while the GFN scores exceeded the cut‐off threshold. This suggested that the machine learning strategy implemented in our study was able to reveal non‐linear co‐functional associations, which could be neglected based on previously established methods.^15^

### 3.4 *β*‐catenin targets significantly associated with glioma prognosis

**FIGURE 6:**
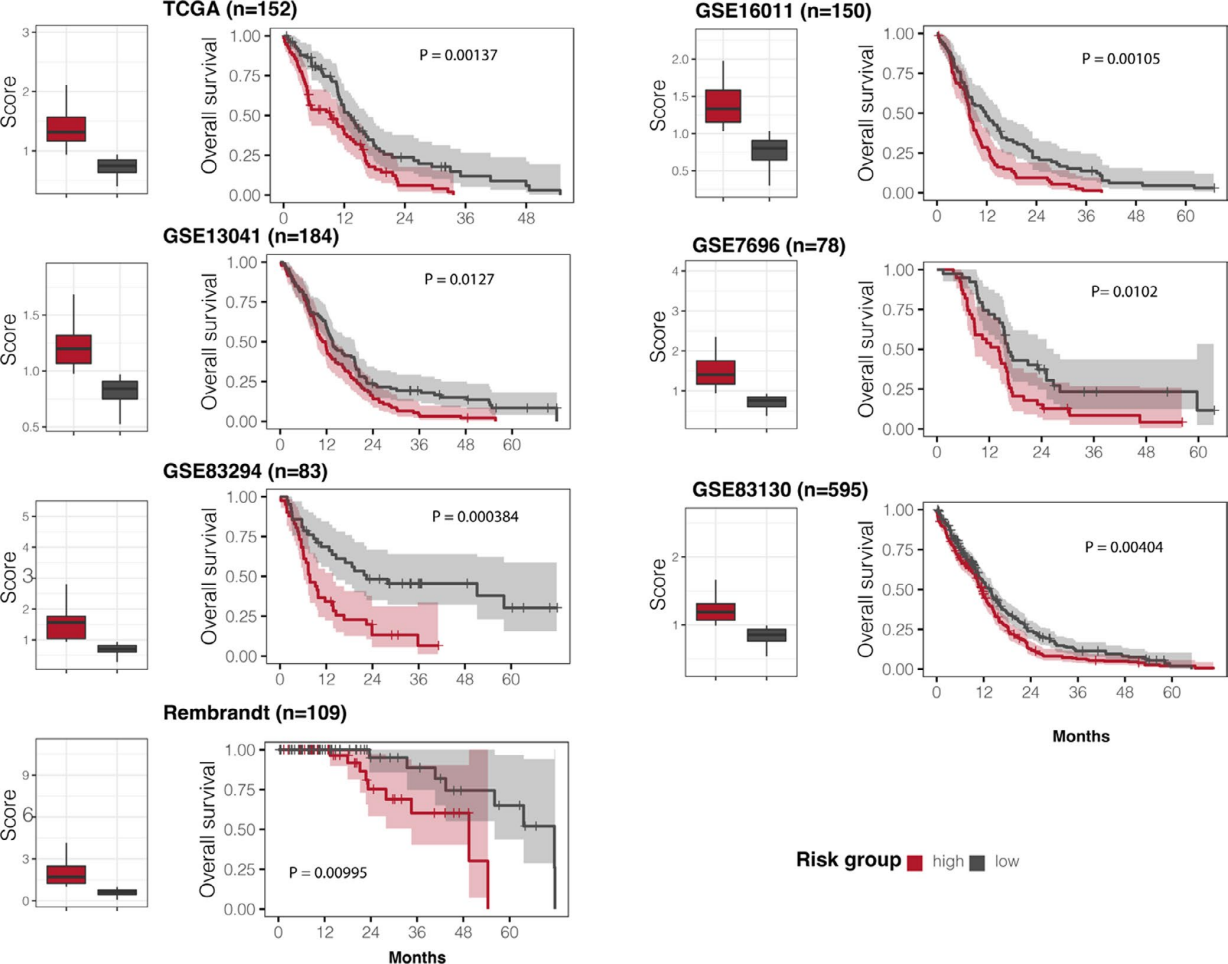
Survival analysis of *β*‐catenin target genes in four glioma studies. Left panel: samples were divided into high‐ and low‐risk groups based on the risk scores generated from prognostic modelling using expression levels of *β*‐catenin target genes. Right panel: Kaplan‐Meier estimated the two groups associated with overall survival. P value was computed using the log‐rank test

While *β*‐catenin dysregulations may involve in glioma progressions, we sought to fit expression levels of the predicted *β*‐catenin targets in the proportional hazards regression models^34^ using multivariate analysis. The samples were divided into high‐ and low‐risk groups with a cut‐off of median value of the prognostic risk scores (Figure 6). The established models from *β*‐catenin target signature could successfully distinguish glioma patients in seven independent cohorts: TCGA (*p* = 0.00137), GSE13041 (*p* = 0.0127), GSE83294 (*p* = 0.000384), GSE68848 (*p* = 0.00995), GSE16011 (*p* = 0.00105), GSE7696 (*p* = 0.0102) and GSE83130 (*p* = 0.00404) (Figure 6).

### 3.5 Identification of a glioblastoma neoplastic cell marker from GFN modules

**FIGURE 7:**
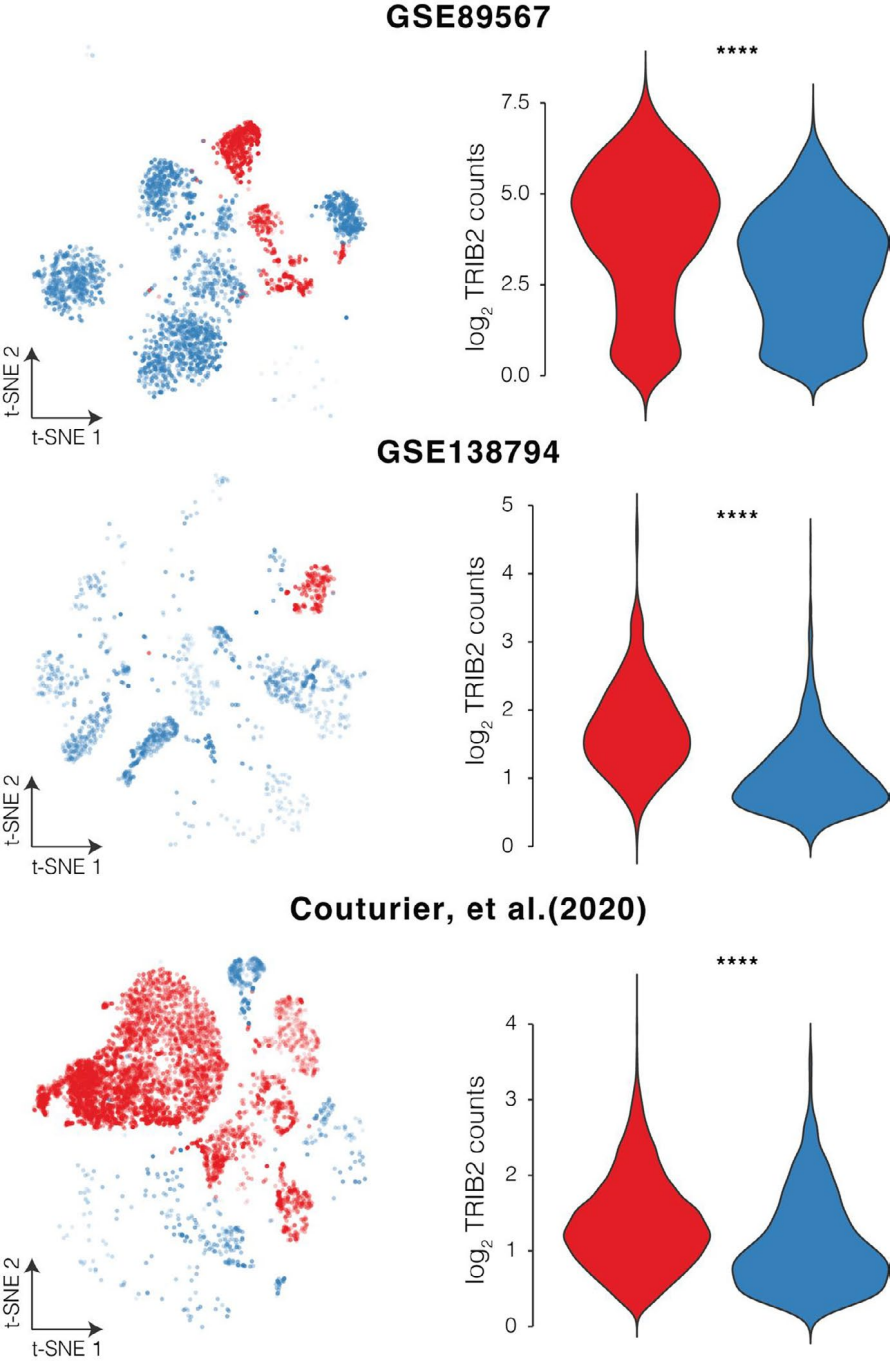
Identification of a glioblastoma neoplastic cell marker from single‐cell RNA‐seq of GBM samples. Left panel: t‐SNE plots showing expression levels. The opacity of each dot represents the level to what extent the corresponding gene is expressed in the cell. Right panel: Violin plots showing distributions of express levels of the corresponding genes in each cell type. (****p < 0.00001, by Wilcoxon rank sum and signed rank tests)

For decades, great challenges remained in glioblastoma treatment due to tumour heterogeneity.^67^ Nevertheless, the recent advancing single‐cell RNA‐seq technologies helped reveal gene expression profiles from gliomas at single‐cell resolution.^37^ From GFN modules, we identified one glioblastoma neoplastic cell marker, TRIB2 (Tribbles Pseudokinase 2) from CID‐40. Typically, neoplastic cells originated from tumour cores and exhibited high expression levels in EGFR and SOX9.^37^ As shown in Figure 7, TRIB2 exhibited 1.38–2.22 fold‐change in neoplastic cells compared to non‐neoplastic cells (*p* < 4.53 × 10^−26^). TRIB2 was previously identified as an important oncogene in lung cancer,^68^ liver cancer^69^ and colorectal cancer.^70^ For gliomas, one recent study demonstrated that its combined elevated expression with MAP3K1 was significantly associated with survival and chemoresistance.^71^ Our study showed specificity of TRIB2 expression in glioblastoma neoplastic cells. While these data were at single‐cell resolution and the elevated patterns are consistent in three independent cohorts, this could shed light on the possibility of becoming a drug target.

## 4 DISCUSSION

In this study, we presented a systems biology approach to identify GFN by applying machine learning algorithms on multiple similarities of genome‐wide fitness screening data. We demonstrated the networks involved in glioma tumorigenesis and predicted potential targets of WNT/*β*‐catenin pathways. These targets are significantly associated with glioma overall survival prognosis and also could be used as cell type‐specific markers for the scRNA‐seq data analysis. While most gene co‐functional associations have not been reported before, they were significantly enriched biological pathways. This could serve a novel resource for studying tumour biology in gliomas. Additionally, we have demonstrated our computational strategy could capture gene co‐functional associations that may be lost in previously established methods.^15^ We reasoned that functionally associated gene pairs may share similar fitness patterns in a non‐linear manner, which could be utilized by machine learning algorithms. This has provided another approach to identify gene co‐functional associations in addition to linear methods, such as PCC and PCA.

From the GFN, we further identified a total of 88 glioma functional modules, which are densely connected in the GFN. Consistent with previous findings,^15^ these modules are significantly enriched in biological pathways, or protein complexes. From one of the modules, we predicted *β*‐catenin targets from its direct functional associations. Statistical modelling of expression levels of these targets was significantly associated with glioma overall survival prognosis. However, these findings need to be verified in ChIP‐seq for binding sites and loss‐of‐function experiments. Lastly, we showed the identification of a glioma neoplastic cell marker, TRIB2, from single‐cell RNA‐seq data analysis, which could potentially become a drug target to tackle tumour heterogeneity challenges.

Taken together, we anticipate that the outcome of this study will significantly advance the understanding of tumour biology and the molecular attributes of glioma progression, but also facilitate the development of diagnostic assays for clinical applications as a complementary to the traditional histopathological assessments. We have also demonstrated the powerful capacity of the systems biology approach implemented in this project to elucidate biomarkers in various types of cancer. As the wealth of multi‐omics data grows, the robustness of biomarkers could be improved by optimizing data from various sources, which could be expanded to a wider range of aspects, such as drug repurposing and personalized treatments in cancer.

## CONFLICT OF INTEREST

Not applicable.

## AUTHOR CONTRIBUTION

Chun‐xiang Xiang: Conceptualization (equal); Formal analysis (equal); Software (lead). Xi‐guo Liu: Conceptualization (equal); Formal analysis (equal). Da‐quan Zhou: Formal analysis (supporting); Project administration (supporting). Yi Zhou: Data curation (lead); Formal analysis (supporting); Resources (lead). Xu Wang: Conceptualization (equal); Formal analysis (equal). Feng Chen: Conceptualization (lead); Project administration (lead).

## PATIENT CONSENT FOR PUBLICATION

Not applicable.

## Supporting information

Table S1

Table S2

## Data Availability

Not applicable.
